# Therapeutic Efficacy of Human Embryonic Stem Cell-Derived Multipotent Stem/Stromal Cells in Diabetic Detrusor Underactivity: A Preclinical Study

**DOI:** 10.3390/jcm9092853

**Published:** 2020-09-03

**Authors:** Jung Hyun Shin, Chae-Min Ryu, Hyein Ju, Hwan Yeul Yu, Sujin Song, Ki-Sung Hong, Hyung-Min Chung, Juhyun Park, Dong-Myung Shin, Myung-Soo Choo

**Affiliations:** 1 Department of Urology, Asan Medical Center, University of Ulsan College of Medicine, Seoul 05505, Korea; jshin@amc.seoul.kr (J.H.S.); uropark@naver.com (J.P.); 2 Department of Biomedical Sciences, Asan Medical Center, University of Ulsan College of Medicine, Seoul 05505, Korea; chaemin0427@hanmail.net (C.-M.R.); alal0903@naver.com (H.J.); hwanyel@naver.com (H.Y.Y.); thdtnwls16@naver.com (S.S.); 3 Department of Stem Cell Biology, School of Medicine, Konkuk University, Seoul 05029, Korea; gorebush@hanmail.net (K.-S.H.); stem_chung@naver.com (H.-M.C.)

**Keywords:** mesenchymal stem/stromal cell, detrusor underactivity, streptozotocin, apoptosis

## Abstract

Mesenchymal stem/stromal cell (MSC) therapy is a promising approach for treatment of as yet incurable detrusor underactivity (DUA), which is characterized by decreased detrusor contraction strength and/or duration, leading to prolonged bladder emptying. In the present study, we demonstrated the therapeutic potential of human embryonic stem cell (ESC)-derived multipotent MSCs (M-MSCs) in a diabetic rat model of DUA. Diabetes mellitus (DM) was induced by intraperitoneal injection of streptozotocin (STZ) (50 mg/kg) into 8-week-old female Sprague-Dawley rats. Three weeks later, various doses of M-MSCs (0.25, 0.5, and 1 × 10^6^ cells) or an equivalent volume of PBS were injected into the outer layer of the bladder. Awake cystometry, organ bath, histological, and gene expression analyses were evaluated 1 week (short-term) or 2 and 4 weeks (long-term) after M-MSC transplantation. STZ-induced diabetic rats developed DUA, including phenotypes with significantly longer micturition intervals, increased residual urine amounts and bladder capacity, decreased micturition pressure on awake cystometry, and contractile responses to various stimuli in organ bath studies. Muscle degeneration, mast cell infiltration, fibrosis, and apoptosis were present in the bladders of DM animals. A single local transplantation of M-MSCs ameliorated DUA bladder pathology, including functional changes and histological evaluation, and caused few adverse outcomes. Immunostaining and gene expression analysis revealed that the transplanted M-MSCs supported myogenic restoration primarily by engrafting into bladder tissue via pericytes, and subsequently exerting paracrine effects to prevent apoptotic cell death in bladder tissue. The therapeutic efficacy of M-MSCs was superior to that of human umbilical cord-derived MSCs at the early time point (1 week). However, the difference in efficacy between M-MSCs and human umbilical cord-derived MSCs was statistically insignificant at the later time points (2 and 4 weeks). Collectively, the present study provides the first evidence for improved therapeutic efficacy of a human ESC derivative in a preclinical model of DM-associated DUA.

## 1. Introduction

Detrusor underactivity (DUA) is a urodynamic disorder defined as “a contraction of reduced strength and/or duration, resulting in prolonged bladder emptying and/or failure to achieve complete bladder emptying within a normal time span” by the International Continence Society [1]. The actual prevalence of DUA is unknown, as its diagnosis is based on invasive pressure flow studies, but DUA is thought to be more common in the elderly [2]. DUA causes both storage and voiding lower urinary tract symptoms, recurrent urinary tract infection, and urinary retention, resulting in deterioration of patient quality of life [3]. Recently, the concept of underactive bladder (UAB) has been introduced for symptom-based correlation with DUA [4].

Aging, neurological deficits, diabetes mellitus (DM), and persistent bladder outlet obstruction with consequent detrusor hypertrophy are the primary risk factors for DUA [5]. Among these DUA etiologies, DM is one of the most important global public health challenges of the 21st century, with an estimated incidence of up to 10% [6]. DM is a predisposing factor for various complications, and diabetic cystopathy occurs in 50% of persons with diabetes [7]. The duration, severity, and management of the disease affect the severity of diabetic cystopathy [8]. The exact pathogenesis of diabetic cystopathy is unclear, but it is suggested that myogenic dysfunction of the detrusor leads to decreased contractility, and that DM-associated autonomic neuropathy decreases bladder sensation [9,10]. Treatment modalities for DUA can be subcategorized into interventions that augment detrusor contractility, reduce bladder outlet resistance, and circumvent existing problems with catheterization [11]. However, effective pharmacological approaches and surgical interventions for DUA are lacking [12], with up to 84% of DUA patients that initially choose conservative management remaining untreated over a nearly 14-year follow-up [13].

Accumulating evidence from preclinical and clinical studies has demonstrated that stem cell therapy is beneficial in the treatment of various bladder dysfunction disorders [14,15,16]. Due to the unique properties of mesenchymal stem/stromal cells (MSCs), such as self-renewal, differentiation, and migration capacity to damaged tissues, MSCs have been considered as a potential stem cell source for regeneration of damaged tissues in a wide range of intractable disorders [17,18,19]. Furthermore, MSCs secrete a variety of trophic factors with immunomodulatory and tissue regenerative properties, accelerating the tissue repair process. MSCs have been isolated from adult tissues, including bone marrow, adipose tissue, and dental pulp, as well as from fetal tissues, including umbilical cord (UC) tissue, UC blood, and placental tissue [20]. However, knowledge of the efficacy and safety of MSCs derived from adult or fetal tissues is currently insufficient for routine clinical application of these modalities. Common deficits include unexpected functional loss following ex vivo stem cell expansion, and limited understanding of the underlying mechanisms of action [20]. Recently, direct derivation of MSCs from pluripotent stem cells (PSCs), such as embryonic stem cells (ESCs) [21,22] and induced PSCs (iPSCs) [23], has been identified as an effective alternative to obtain sufficient numbers of progenitor cells for use in cell therapies or regenerative medicine.

Previously, we demonstrated the superior therapeutic efficacy of multipotent MSCs (M-MSCs) derived from human ESCs compared with adult tissue-derived MSCs in animal models representing different pathologies of interstitial cystitis/bladder pain syndrome (IC/BPS) [24,25,26]. In the present preclinical study, we examined the therapeutic efficacy of M-MSCS in DM-associated DUA, and assessed the underlying mechanisms of action.

## 2. Materials and Methods

### 2.1. Study Approval

All animal studies were approved by the Institutional Animal Care and Use Committee of the University of Ulsan College of Medicine (IACUC-2016-12-088). Human UC samples were obtained from healthy, normal, full-term newborns after obtaining written informed parental consent in accordance with the guidelines approved by the Ethics Committee on the Use of Human Subjects at Asan Medical Center. Informed consent was obtained from all pregnant mothers prior to UC collection.

### 2.2. MSC Cell Culture and Establishment of GFP^+^ M-MSCs

M-MSCs differentiated from human H9 ESCs [21] were maintained in EGM2-MV medium (Lonza, San Diego, CA, USA) on rat tail collagen type I (Sigma-Aldrich, St. Louis, MO, USA)-coated plates in a humidified and heated atmosphere of 5% CO_2_ and 37 °C as previously described [24,26]. Human UC-MSCs were cultured in low-glucose DMEM containing 10% heat-inactivated FBS, 5 ng/mL human epidermal growth factor (Sigma-Aldrich, St. Louis, MO, USA), 10 ng/mL basic fibroblast growth factor, and 50 ng/mL long-R3 insulin-like growth factor-1 (ProSpec, Rehovot, Israel) as previously described [27]. Both M-MSCs and UC-MSCs were positive for the expression of CD29, CD73, and CD105 surface molecules but lacked CD14, CD34, and CD45 hematopoietic lineage marker expression (Supplementary Figure S1). All M-MSCs were expanded for less than ten passages to ensure multipotency. To induce stable GFP expression, M-MSCs were infected with a GFP-expressing lentivirus, which was generated as described previously [28,29]. M-MSCs were infected with concentrated lentivirus containing a GFP expression construct using 6 µg/mL polybrene (Invitrogen, Carlsbad, CA, USA), and infected cells were selected using 6 µg/mL blasticidin (Invitrogen).

### 2.3. Animal Model and Study Design

Female Sprague-Dawley rats (8 weeks old) were used in this study. streptozotocin (STZ) (Sigma Chemical Company, St. Louis, MO, USA) was used to induce type I diabetes. After fasting overnight, rats were injected intraperitoneally with STZ (50 mg/kg) dissolved in 0.1 M citrate acid buffer (pH 4.5) solution. For the non-diabetic control group, rats were injected with an equivalent volume of citrate buffer solution. Blood glucose level was measured 72 h later using samples obtained by tail prick. Rats with blood glucose higher than 200 mg/dL (16.7 mmoL/L) were selected as diabetic rats and used for subsequent studies. Three weeks after induction of diabetes, diabetic rats were anesthetized with 0.2 mL tiletamine (Zoletil1; Virbac Laboratories, Carros, France) for stem cell transplantation. To evaluate the therapeutic efficacy of M-MSCs, the rats were distributed into five groups: non-diabetic control (sham), untreated diabetic (DM), and diabetic rats that received a single administration of the indicated dosage of human ESC-derived M-MSCs (0.25, 0.5, and 1 × 10^6^ cells; DM groups + 250 K, 500 K, 1000 K) resuspended in 200 µL phosphate buffered saline (PBS). For non-diabetic and untreated DM groups, PBS was injected instead of stem cells. PBS and M-MSCs were directly injected into the outer layer (serosa) of the anterior wall of the bladder as previously described [24,26]. Human UC-MSCs were transplanted using the same procedure. The therapeutic effect of stem cell transplantation was examined using awake cystometry and organ bath evaluation, as well as histological and gene expression analysis 1 week (short-term) or 2 and 4 weeks (long-term) after stem cell injection.

### 2.4. Evaluation of Bladder Function and Tissue Preparation

Cystometric evaluation was performed in conscious unrestrained animals in metabolic cages. Three days prior to the cystometrogram, intravesical pressure (IVP) and intra-abdominal pressure (IAP) were measured. The urethra was approached using a PE-50 catheter (Clay Adams, Parsippany, NJ, USA) connected to a pressure transducer (Research Grade Blood Pressure Transducer; Harvard Apparatus, Holliston, MA, USA) and a microinjection pump (PHD22/2000 pump; Harvard Apparatus). Voiding volumes were recorded by means of a fluid collector connected to a force displacement transducer (Research Grade Isometric Transducer; Harvard Apparatus) as normal saline was infused into the bladder at a rate of 0.4 mL/min. IVP, IAP, and voiding volume were recorded continuously using Acq Knowledge 3.8.1 software and an MP150 data acquisition system (Biopac Systems, Goleta, CA, USA) at a sampling rate of 50 Hz. The mean values from three reproducible voiding cycles of each individual animal were used for the analysis. Non-voiding contractions (NVCs) were considered to have occurred when the IVP increments exceeded 15 cm H_2_O from baseline in the absence of expelled urine. Bladder pressure (BP) was defined as the lowest bladder pressure during filling; micturition pressure was defined as the maximum BP during the micturition cycle; micturition volume (MV) was defined as the volume of expelled urine; and residual volume (RV) was defined as the urine volume remaining after voiding. Consistent with prior studies, the micturition interval (MI) was defined as the interval between micturition contraction cycle, representing the times between resting to basal pressure in the preceding micturition cycle and rising from basal pressure in the following cycle. Bladder capacity (BC) was defined as MV + RV. Bladder voiding efficiency (BVE) was defined as 100 × MV/BC. The mean values from three reproducible micturition cycles were evaluated in five individual animals per group. After evaluation of voiding function, rat bladders were harvested. Half of each bladder was cryopreserved in liquid nitrogen for RNA isolation. The remaining half was fixed in 4% buffered formalin and embedded in paraffin for histological examination or immunohistochemical staining.

### 2.5. Histological Analysis and Immunofluorescent Staining

After 24 h of fixation in 4% paraformaldehyde, bladders were embedded in paraffin, cut on a microtome into 3 µm slices, affixed to slides, and stained with hematoxylin and eosin (H&E). Histological quantification of smooth muscle, tissue fibrosis, and apoptosis were conducted using Masson’s trichrome staining (Junsei Chemical, Tokyo, Japan), toluidine blue staining (8544–4125; Daejung Chemicals & Metals, Seoul, Korea), and TUNEL staining (1 684 795; Roche, Mannheim, Germany), respectively.

The engraftment and cellular fate of GFP^+^ M-MSCs transplanted into bladders were examined by immunofluorescent staining with antibodies specific to GFP (ab290; Abcam, Cambridge, MA, USA), α-SMA (ab7817; Abcam, Cambridge, MA, USA), Desmin (sc-23879; Santa Cruz Biotechnology, Dallas, TX, USA), vimentin (sc-6260; Santa Cruz Biotechnology, Biotechnology, Dallas, TX, USA), NG2 (sc-53389; Santa Cruz Biotechnology, Dallas, TX, USA), caspase 7 (sc-56063; Santa Cruz Biotechnology, Dallas, TX, USA), and CD31 (sc-376764; Santa Cruz Biotechnology, Dallas, TX, USA). Alexa Fluor 488-conjugated (A11001) or Alexa Fluor 564-conjugated (A11010) anti-mouse or anti-rabbit antibodies were used as secondary antibodies (Molecular Probes, Grand Island, NY, USA). Nuclei were counterstained with 4′,6-diamino-2-phenylindole (DAPI; D9542; Sigma-Aldrich, St. Louis, MO, USA). Proliferation of human cells transplanted into bladders was determined by immunohistochemistry analysis using an antibody specific to human Ki67 (mouse monoclonal, M7240; Dako, Santa Clara, CA, USA). For immunohistochemistry studies of engrafted bladder tissue, three representative areas were randomly selected from five separate animals per group and the mean values from the selected areas for each individual animal were used for the analysis. Quantitative digital image analysis was performed using Image Pro 5.0 software (Media-Cybernetics, Rockville, MD, USA).

### 2.6. Gene Expression Analyses

Total RNA was isolated using a RNeasy Mini Kit (Qiagen Inc., Valencia, CA, USA). Reverse transcription was performed using TaqMan Reverse Transcription Reagents (Applied Biosystems, Foster City, CA, USA). The indicated transcripts were quantified by real-time quantitative PCR (RQ-PCR) using a PikoReal Real-Time PCR System (Thermo Scientific, Foster City, CA, USA) and iQ SYBR Green PCR Master Mix (Bio-Rad, Hercules, CA, USA), as described previously [30,31]. The threshold cycle (Ct) of the indicated genes was subtracted from that of *Gapdh*, an endogenous control gene, and the expression level of the target genes was quantified as 100% × 2^−∆Ct(target-*Gapdh*)^, representing the percentage of *Gapdh*. Duplicate RQ-PCR assays were performed using five separate animals per group and the mean values from each individual animal were used for the analysis.

### 2.7. Organ Bath Study

Bladders (*n* = 5 animals/group) were cut into two strips with the mucosa along the longitudinal axis. The strips were mounted in an organ bath system (Danish Myo Technology, Aarhus, Denmark) containing 15 mL Krebs buffer. Bladder strips were subjected to a resting tension of 1 g and allowed to stabilize for at least 60 min. Contractions were recorded as changes in bladder strip tension from baseline in response to 80 mM KCl, a concentration gradient of carbachol (3–100 mM), electrical field stimulation (EFS; 1, 2, 4, 8, 16, and 32 Hz), and 1 mM ATP. All tissue responses (g) were normalized to tissue weight (g tissue) for the analysis (g/g tissue). Drug concentrations are expressed as final concentration in the organ bath.

### 2.8. Statistical Analysis

Data are expressed as means ± standard error of the mean (SEM), and were analyzed using GraphPad Prism 7.0 software (GraphPad Software, La Jolla, CA, USA). Statistical significance was assessed using a one-way or two-way ANOVA followed by Bonferroni post-hoc tests. A *p*-value < 0.05 was considered statistically significant. Source data with the exact experimental values, number of replicates, and statistical test results can be found in the Supplementary Table.

## 3. Results

### 3.1. Therapeutic Efficacy of M-MSCs in an Animal Model of Diabetic DUA

**Figure 1:**
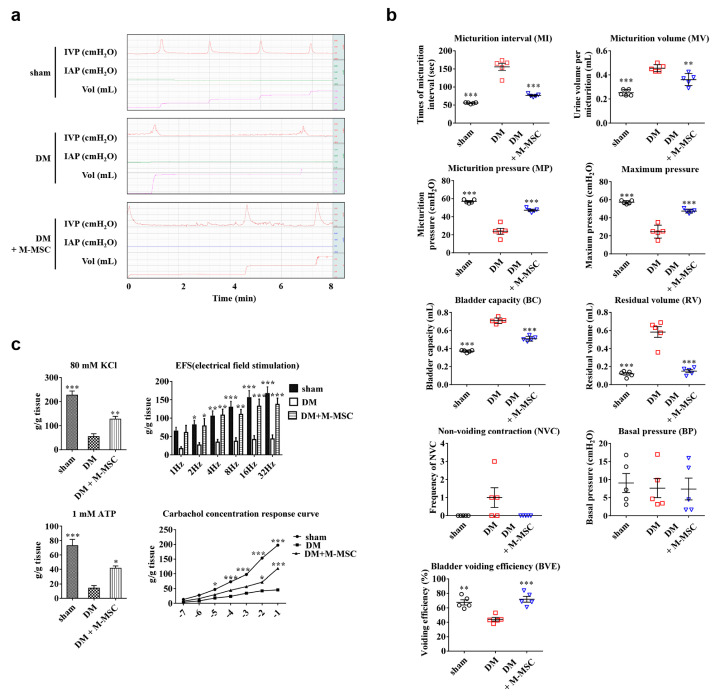
M-MSC transplantation ameliorated voiding function in DM rats. (**a**) Representative awake cystometry results and (**b**) quantitative voiding data 1 week after injection of diabetes mellitus (DM) rats with 1 × 10^6^ M-MSCs (1000 K) from five independent animals per group. Sham: non-diabetic sham-operated. (**c**) Organ bath study analysis (n = 5 animals/group) to assess contractile response to 80 mM KCl, frequency response to EFS, contractile response to 1 mM ATP, and concentration response curve for carbachol. Data are presented as mean ± SEM. (* *p* < 0.05, ** *p* < 0.01, and *** *p* < 0.001 relative to DM group, one-way or two-way ANOVA with Bonferroni post-hoc comparison). The exact experimental and statistical values can be found in the Supplementary Table. DM: diabetes mellitus; M-MSC: Multipotent-mesenchymal stem cell; EFS: Electrical field stimulation.

M-MSCs (1 × 10^6^ cells) were transplanted via a single engraftment to the bladder in the diabetic DUA rat model. Subsequently, awake filling and voiding cystometrogram studies were conducted, allowing long-term evaluation of bladder voiding function in live, free-moving animals [24,25,26]. DM rats exhibited DUA-like voiding patterns (Figure 1a,b), characterized by increased MI in DM animals (155.5 ± 9.87 sec in DM vs. 55.51 ± 0.86 sec in non-diabetic; *p* < 0.001) and increased MV (0.46 ± 0.01 vs. 0.25 ± 0.01 mL; *p* < 0.001). Further, DM animals exhibited decreased micturition pressure (23.85 ± 3.15 vs. 56.98 ± 0.87 cm H_2_O; *p* < 0.001) and decreased maximum pressure (24.61 ± 3.2 vs. 57.15 ± 0.85 cm H_2_O; *p* < 0.001). DM animals also exhibited increased BC (0.71 ± 0.01 vs. 0.37 ± 0.01 mL; *p* < 0.001) and increased RV (0.58 ± 0.06 vs. 0.12 ± 0.01 mL; *p* < 0.001), but decreased BVE (44.16 ± 2.46 vs. 67.51 ± 3.64 mL; *p* < 0.01). Importantly, these defects in voiding parameters were significantly ameliorated in the M-MSC injected DM group (Figure 1a,b).

We next examined the overall contractile response in an organ bath study. Consistent with the awake cystometry results, bladder strips from the DM group exhibited significant defects in the contractile responses to 80 mM KCl, 1 mM ATP; a defective frequency response to EFS; and an impaired concentration response curve to carbachol (Cch) relative to non-diabetic animals. M-MSC therapy significantly restored defects in contractile responses to these stimuli (Figure 1c).

### 3.2. Long-Term Therapeutic Effects of M-MSC Transplantation

**Figure 2:**
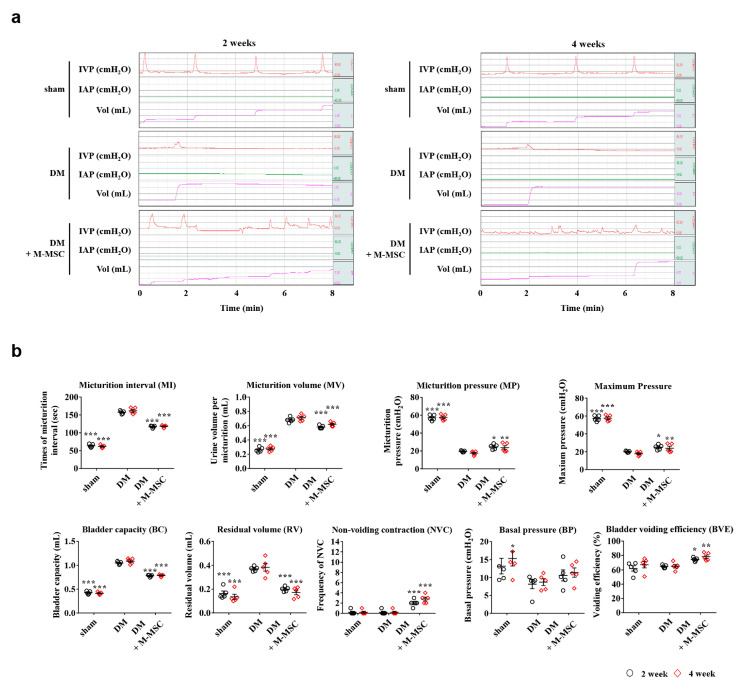
Long-term therapeutic effects of M-MSCs in DM rats. (**a**) Representative awake cystometry results (**b**) and quantitative bladder voiding data in DM rats at 2 and 4 weeks after injection of 1 × 10^6^ M-MSCs. Data are presented as mean ± SEM (*n* = 5, * *p* < 0.05, ** *p* < 0.01 and *** *p* < 0.001 relative to DM group, two-way ANOVA with Bonferroni post-hoc comparison). The exact experimental and statistical values can be found in the Supplementary Table. DM: diabetes mellitus; M-MSC: Multipotent-mesenchymal stem cell.

In our previous study of an IC/BPS rat model, the therapeutic effects of a single M-MSC administration on bladder voiding function were sustained for 2–4 weeks following transplantation [26]. Therefore, we examined the long-term therapeutic effects of M-MSCs in the diabetic DUA model by measuring bladder voiding parameters using awake cystometry 2 and 4 weeks after a single administration of 1 × 10^6^ M-MSCs. The DM group presented a gradually elongated MI, larger RV, and decreased micturition. In contrast to the short-term (1-week) follow-up, M-MSC transplantation did not substantially restore DUA voiding phenotypes in either the 2- or 4-week long-term follow-ups (Figure 2a,b). In the long-term follow-ups, minimal effects were found in the BVE in all tested groups (Figure 2b).

### 3.3. Determining Optimal Cell Dosage for M-MSC Therapy

**Figure 3:**
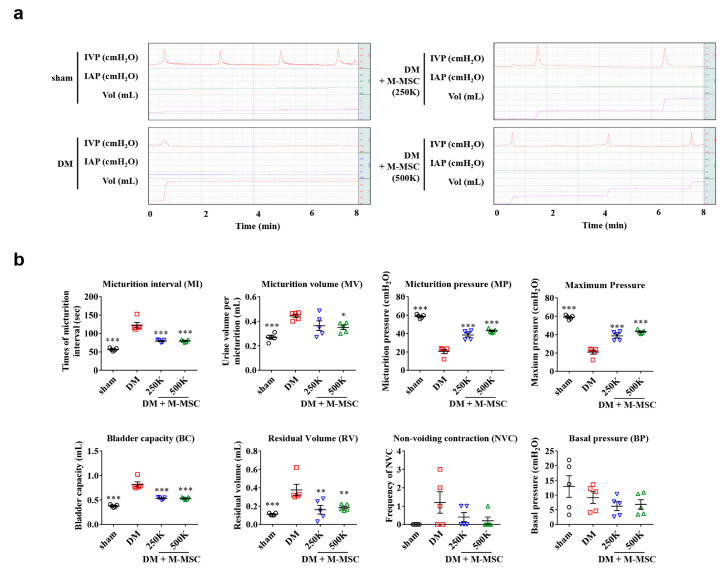
Dose-dependent effects of M-MSCs on bladder function. (**a**) Representative awake cystometry results and (**b**) quantitative voiding data 1 week after injection of 2.5 × 10^5^ (250 K) or 5.0 × 10^5^ (500 K) M-MSCs into the bladders of DM rats. Data are presented as mean ± SEM (*n* = 5, * *p* < 0.05, ** *p* < 0.01, and *** *p* < 0.001 relative to DM group, one-way ANOVA with Bonferroni post-hoc test comparison). The exact experimental and statistical values can be found in the Supplementary Table. DM: diabetes mellitus; M-MSC: Multipotent-mesenchymal stem cell.

Considering the risk of neoplasia in stem cell therapy, in addition to the time and cost required for isolation and expansion of stem cells, determining the critical cell dosage for therapeutic efficacy is of significant clinical relevance. Therefore, we reduced the transplanted cell numbers to 2.5 × 10^5^ (250 K) and 5.0 × 10^5^ (500 K), and examined the therapeutic efficacies of these dosages in diabetic DUA over the short-term follow-up period. Awake cystometry revealed that the significant beneficial effects of M-MSCs on bladder voiding functions were also conferred by administration of as few as 2.5 × 10^5^ M-MSCs (Figure 3a,b), although lower dosages of M-MSCs had decreased therapeutic effects relative to the 1 × 10^6^ M-MSC group. Therapeutic efficacy did not significantly differ between the 250 K and 500 K groups.

**Figure 4:**
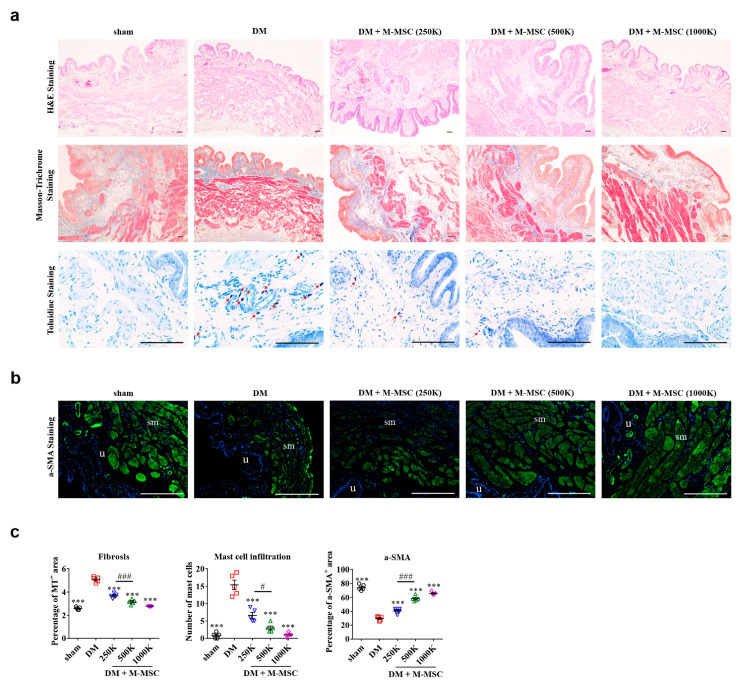
Injection of M-MSCs restored histological abnormalities in DM rat bladders. (**a**) Histological analysis for (i) hematoxylin and eosin (H&E) staining (100× magnification; scale bar, 200 µm), (ii) Masson’s trichrome staining (100× magnification; scale bar, 200 µm), and (iii) toluidine blue staining (400× magnification; scale bar, 100 µm) of bladder tissues from DM rats 1 week after injection of the indicated number of M-MSCs or PBS. (**b**) Immunohistochemical analysis of α-SMA staining (400× magnification; scale bar, 100 µm) of bladder tissues from the indicated groups. Arrows indicate infiltrated mast cells. Sham: non-diabetic sham-operated. (**c**) Quantification of histological staining. Three representative areas per slide were randomly selected from five individual animals per group. Data were normalized against sham-operated rats and are presented as mean ± SEM (*n* = 5, *** *p* < 0.001, relative to DM group; # *p* < 0.05 and ### *p* < 0.001 relative to 500 K group, one-way ANOVA with Bonferroni post-hoc comparison). The exact experimental and statistical values can be found in the Supplementary Table. DM: diabetes mellitus; M-MSC: Multipotent-mesenchymal stem cell.

To further compare the dose-dependent effects of M-MSC transplantation, we evaluated the severity of pathologies present in diabetic DUA patients. Histological examinations revealed that severe muscle atrophy, tissue fibrosis, and infiltration of toluidine blue-stained mast cells were present in DM bladders (Figure 4a,c). Smooth muscle atrophy was also demonstrated by both Masson’s trichrome staining and immunofluorescent staining for α-SMA^+^ cells in the bladder (Figure 4b,c). Transplantation of as few as 2.5 × 10^5^ M-MSCs significantly improved the detrusor muscle damage, fibrosis, and mast cell infiltration observed in DM rat bladders. Importantly, therapeutic efficacy was dependent on the dose of transplanted cells (Figure 4c), with at least 5.0 × 10^5^ M-MSCs necessary to confer significant therapeutic effects. These histological findings, together with the awake cystometry results, demonstrated that a single administration of 5.0 × 10^5^ M-MSCs into the bladder was the optimal cell dosage for treatment of diabetic DUA.

### 3.4. Therapeutic Efficacy of M-MSCs Relative to Umbilical Cord-Derived Stem Cells

**Figure 5:**
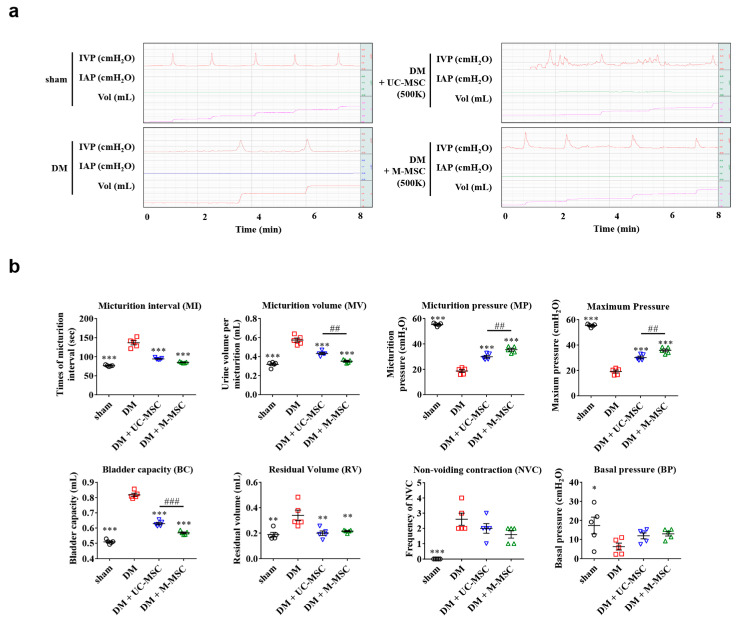
Superior efficacy of M-MSCs to UC-MSCs. (**a**) Representative awake cystometry results and (**b**) quantitative voiding data 1 week after injection of M-MSCs or UC-MSCs (0.5 × 10^6^ cells; 500 K) into the bladders of DM rats. All data are presented as the mean ± SEM (*n* = 5). Data were analyzed using a one-way ANOVA with Bonferroni post-hoc comparison (* *p* < 0.05, ** *p* < 0.01 and *** *p* < 0.001 relative to DM group; ## *p* < 0.01, ### *p* < 0.001 M-MSC vs. UC-MSC groups). The exact experimental and statistical values can be found in the Supplementary Table. DM: diabetes mellitus; M-MSC: Multipotent-mesenchymal stem cell.

We next compared the therapeutic efficacy of M-MSCs by transplanting either 5.0 × 10^5^ M-MSCs or 5.0 × 10^5^ human UC-MSCs into DM animals, followed by short-term (1-week) follow-up. Awake cystometry revealed that administration of UC-MSCs also decreased MI, increased micturition pressure, and decreased post-void RV in DM animals (Figure 5a,b). However, animals that received UC-MSC transplantation exhibited more frequent NVCs relative to animals that received M-MSC therapy, suggesting that M-MSCs offered superior stabilization of intravesical pressure (Figure 5a,b). Therefore, transplantation of M-MSCs conferred more potent and stable therapeutic outcomes than UC-MSCs.

### 3.5. Cellular Properties of Transplanted M-MSCs

**Figure 6:**
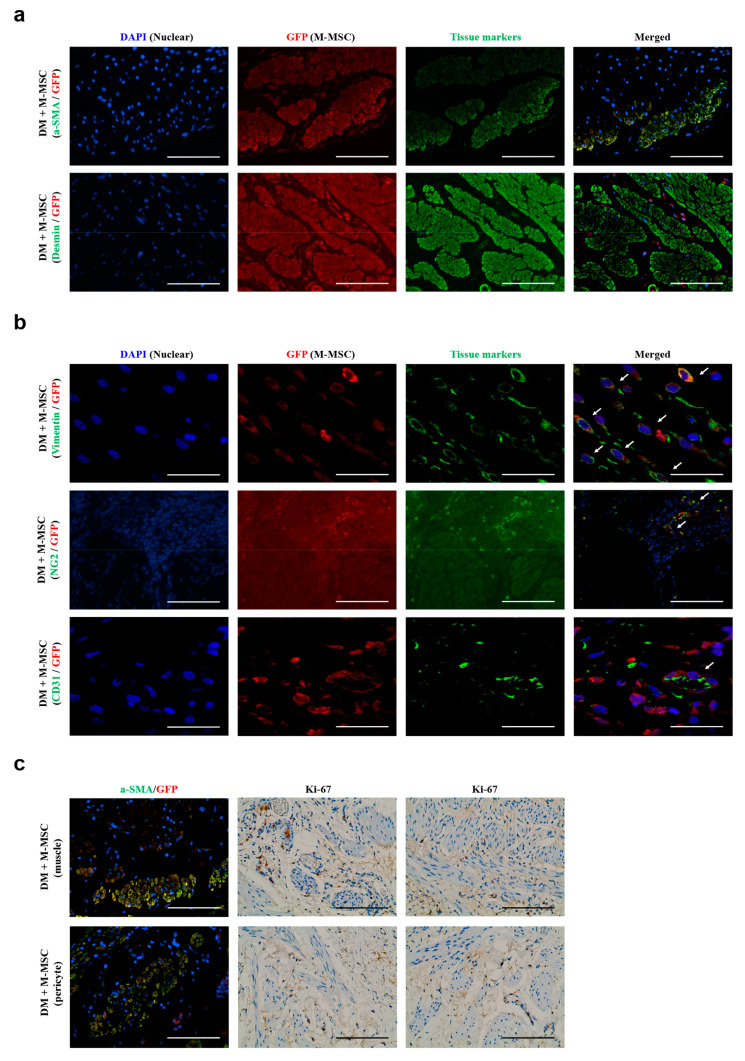
Cellular fate of transplanted M-MSCs. Co-immunofluorescence staining of M-MSCs stably expressing GFP (red) with (**a**) muscle-specific markers (α-SMA and desmin) and (**b**) pericyte-specific markers (vimentin and NG2) and an endothelial marker (CD31) in bladder sections of DM + M-MSC (1000 K) rats at 1 week post-transplant (400× magnification; scale bar, 100 µm). Arrows indicate GFP^+^ engrafted cells co-expressing each tissue marker. (**c**) Proliferation status of engrafted M-MSCs was examined by immunohistochemical staining for the human specific Ki-67 marker of cell proliferation (400× magnification; scale bar, 100 µm). Nuclei were stained with DAPI (blue, (**a**,**b**)) or Mayer’s hematoxylin (**c**). Representative images of co-immunofluorescence staining for GFP and α-SMA proteins are depicted in the left panel of the Ki-67 immunohistochemical staining results.

Because M-MSC transplantation significantly restored muscle degeneration, we determined whether engrafted M-MSCs could promote regeneration of the detrusor muscle in DM animals 1 week after GFP^+^ M-MSC transplantation. Co-immunostaining of GFP^+^ cells with two muscle-specific proteins, α-SMA and desmin, revealed that the majority of the engrafted M-MSCs did not robustly express muscle markers, although some GFP^+^/α-SMA^+^ double positive cells were detected proximal to the serosa where M-MSCs were injected (Figure 6a). However, cells with strong GFP signals were frequently detected proximal to the muscular bundle.

In our previous studies of IC/BPS, the majority of transplanted M-MSCs were integrated into pericytes, expressing the pericyte markers vimentin or NG2 [24,26]. Therefore, we further characterized the GFP^+^ transplanted cells by co-staining with the pericyte markers vimentin and NG2. The majority of GFP^+^ cells located between the serosa and muscularis were strongly labeled with both vimentin and NG2 (Figure 6b). In particular, the engrafted GFP^+^ cells proximal to the muscular bundle strongly expressed the pericyte-specific NG2 protein, indicating that implanted M-MSCs differentiated into stromal and perivascular cells, rather than directly into myocytes. Consistent with these results, staining for human specific Ki-67, a cell proliferation marker, was negative in muscle fibers and was only positive in pericytes (Figure 6c). The GFP^+^ cells located in the endothelium slightly overlapped with CD31^+^ endothelial cells (Figure 6b).

### 3.6. Paracrine Effects of M-MSCs for Suppressing Apoptosis in Diabetic DUA

**Figure 7:**
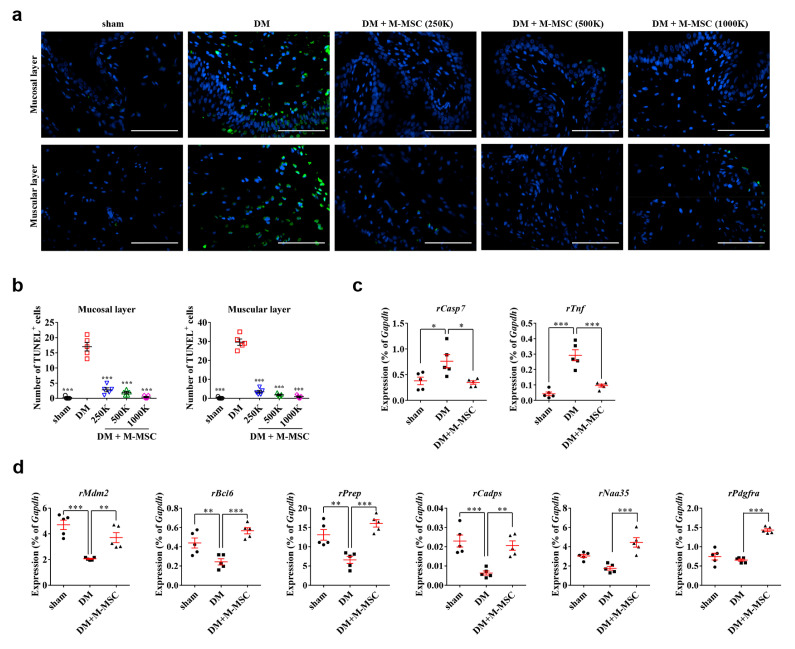
Protective effect of M-MSC therapy against apoptotic cell death in DM bladders. (**a**,**b**) Representative images (400× magnification; scale bar, 100 µm), (**a**,**b**) quantification of TUNEL^+^ cells in bladder tissues of DM rats at 1 week after injection of M-MSCs or PBS. For quantification, three representative areas per slide were randomly selected from five individual animals per group, and the mean values for each animal were presented as mean ± SEM (*n* = 5, ** *p* < 0.01 and *** *p* < 0.001 relative to DM group, one-way ANOVA with Bonferroni post-hoc comparison). (**c**,**d**) RQ-PCR analyses of mRNA levels of (**c**) pro-apoptotic and (**d**) anti-apoptotic genes in bladder tissues of DM rats 1 week after M-MSC or PBS injection. RQ-PCR assays were performed in duplicate for five individual animals per group. Expression levels (% of *Gapdh*) are presented as mean ± SEM (*n* = 5 animals/group). (* *p* < 0.05, ** *p* < 0.01, and *** *p* < 0.001 relative to DM group, one-way ANOVA).

As the engrafted M-MSCs mainly integrated into pericytes rather than directly into myocytes, we examined whether engrafted M-MSCs could support a microenvironment favorable for restoration of detrusor muscle injury. In the bladders of DM animals, TUNEL^+^ apoptotic cells were significantly increased, while injection of as few as 2.5 × 10^5^ M-MSCs ameliorated apoptosis in both the mucosal and muscular layers (Figure 7a,b).

Accordingly, gene expression analysis revealed that M-MSC therapy prevented bladder tissue upregulation of caspase 7 (*Casp7*) and tumor necrosis factor (*Tnf*) mRNA levels in DM bladders (Figure 7c). Immunofluorescence staining confirmed the increase in caspase 7 protein specifically in the DM bladder, but not in the DM bladder with M-MSC transplantation (Supplementary Figure S2). Furthermore, M-MSC therapy increased mRNA levels of anti-apoptotic genes including MDM2 proto-oncogene (*Mdm2*), Bcl6 transcription repressor (*Bcl6*) [32], prolyl Endopeptidase (*Prep*) [33], calcium-dependent secretion activator (*Cadps*) [34], N-alpha-acetyltransferase 35, NatC auxiliary subunit (*Naa35*) [35], and platelet-derived growth factor receptor-alpha (*Pdgfrα*) (Figure 7d). Taken together, these findings suggested that engrafted M-MSCs primarily integrated into pericytes, and could prevent bladder tissue apoptosis in diabetic DUA via paracrine mechanisms.

**Figure 8:**
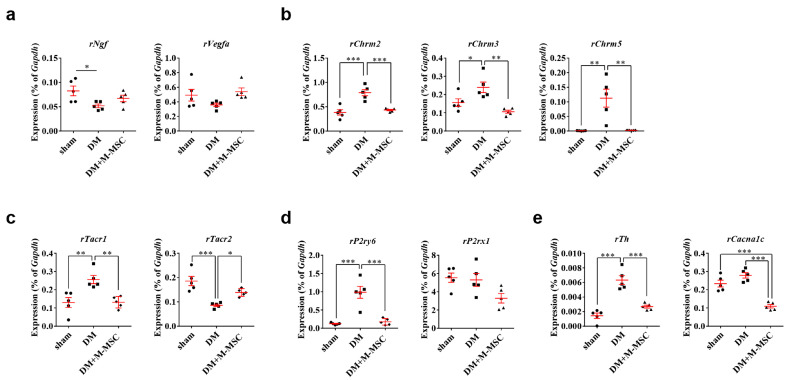
M-MSC therapy restored expression of genes related to diabetic DUA (**a**–**e**) RQ-PCR measurement of mRNA levels for genes related to the pathogenesis of diabetic DUA in the bladders of DM rats at 1 week after injection of M-MSCs or PBS. These genes included (**a**) growth factors such as Nerve growth factor (*Ngf*) and Vascular endothelial growth factor-A (*Vegfa*), (**b**) cholinergic muscarinic receptors, (**c**) tachykinin receptors, (**d**) Pyrimidinergic receptor (*P2ry6*) and Purinergic receptor P2X-1 (*P2rx1*), and (**e**) tyrosine hydroxylase (*Th*) and calcium voltage-gated channel subunit alpha1-C (*Cacna1c*). Expression levels of the indicated transcripts are presented as % *Gapdh*. Expression levels (*n* = 5 animals/group) are presented as means ± SEM. (* *p* < 0.05, ** *p* < 0.01, and *** *p* < 0.001 relative to DM group, one-way ANOVA).

Furthermore, gene expression analysis revealed that M-MSC transplantation significantly restored alternations in gene expression induced by diabetic DUA. Altered genes included (i) growth factors such as nerve growth factor (*Ngf*) and vascular endothelial growth factor-A (*Vegfa*) (Figure 8a), (ii) cholinergic muscarinic receptors (Figure 8b), (iii) tachykinin receptors (Figure 8c), (iv) pyrimidinergic (*P2ry6*) and purinergic receptor P2X-1 (*P2rx1*) (Figure 8d), and (v) tyrosine hydroxylase (*Th*) and calcium voltage-gated channel subunit alpha1-C (*Cacnα1c*) (Figure 8e). In particular, bladder expression of genes associated with mediating contractile stimuli of the detrusor muscle, including cholinergic receptor muscarinic-2 (*Chrm2*), -3 (*Chrm3*), and -5 (*Chrm5*), were increased in the DM group but decreased by M-MSC transplantation (Figure 8b). The effect of M-MSC therapy on gene expression was sustained in the majority of the aforementioned genes at 4 weeks after M-MSC transplantation (Supplementary Figure S3).

## 4. Discussion

In the present study, the STZ-induced diabetic DUA rat model was used to evaluate the therapeutic effects of M-MSCs derived from human ESCs in DM-related DUA. Additionally, we demonstrated that M-MSCs integrated into pericytes and could exert paracrine effects, not only to restore detrusor muscle damage, but also to prevent apoptosis in diabetic bladders.

To our knowledge, this is the first study to demonstrate the therapeutic efficacy of M-MSCs in a diabetic DUA model. In a previous study by Zhang et al., autologous adipose tissue-derived MSCs (ADSCs, 3.0 × 10^6^ cells) were injected via tail vein or directly into bladders. ADSCs significantly ameliorated diabetic bladder dysfunction, and the route of stem cell injection did not affect the therapeutic efficacy of ADSCs [36]. In addition, Zhang et al. suggested that defocused low-energy shock waves recruited endogenous stem cells by increasing secretion of NGF and VEGF [37]. In the present preclinical study, we used a lower dose of M-MSCs derived from human ESCs, and the injection route was via direct bladder transplantation, according to our previously developed protocols in the IC/BPS rat model [24,26].

The advantage of ESCs as an alternative source of MSCs is that ESCs can be indefinitely maintained due to their self-renewal capacity, and at the same time can differentiate into any cell type due to their pluripotency [38,39]. Despite these therapeutic advantages, safety concerns such as the risk of tumorigenesis remain a major obstacle to the therapeutic application of hESC-derived cells [40]. However, the recent successful clinical treatment of ocular disorders with hESC-based therapeutics partly diminished this concern [41,42]. Consistently, our previous 1 year study using a chronic IC/BPS model failed to identify adverse outcomes, including tumorigenesis, abnormal growth, immune reactions, or differentiation of transplanted M-MSCs into unwanted cell types [24,26].

Although the present preclinical diabetic DUA study did not identify any adverse reactions, the safety issues surrounding ESC-based therapies must be thoroughly investigated before this modality can be applied in clinical settings [40]. In addition, the potential for rejection via antigenicity and immunological processes should be carefully considered. T-cells and major histocompatibility complex class 1 (MHC-1) play pivotal roles in the rejection of hESCs and their derivatives [43]. However, prior studies identified that the immune-stimulatory capacity of hESCs and their derivatives is very low [44]. In addition, MSCs derived from iPSCs haven been considered as a potential means to circumvent immune rejection.

Prior studies have demonstrated that iPSC-derived MSCs have therapeutic effects in a wide range of intractable disorders in multiple animal models [23,45,46]. Hyperglycemia can induce cellular damage through oxidative stress and other mechanisms. Excess mitochondrial glucose oxidation produces electrons that generate reactive oxygen species [47]. In vivo experimental studies in animal models identified that iPSC-derived MSCs caused functional changes in detrusor smooth muscle, neuronal impairment, and urothelial dysfunction [48]. Further, the severity of diabetic cystopathy depends primarily on disease duration and management.

DUA or UAB result from various pathological processes that can be classified as idiopathic, neurogenic, or myogenic [49]. Idiopathic DUA is defined as being “free of evident neuropathy, functional or anatomical bladder obstruction and shows low or no detrusor pressure combined with low maximum flow and large post-void residual, or urinary retention” [50]. Neurogenic DUA occurs due to any alteration of neural control mechanisms [51]. Myogenic DUA can result from alterations in ion storage and/or exchange, energy generation, or degenerative changes to bladder smooth muscle [52].

Notably, the pathogenesis of DM-associated bladder DUA is multifactorial with changes in the physiology of detrusor smooth muscle cells, innervation or function of the neuronal component, or urothelial dysfunction [53,54,55]. Detrusor contraction is mediated by acetylcholine released by the pelvic nerve acting on muscarinic receptors. Many neurotransmitters such as adenosine triphosphate, nitric oxide, and prostanoids are released from urothelial cells and these mediators can be increased in response to urothelial injury or inflammation, resulting in a variety of abnormalities in DM-induced bladder dysfunction [55]. Therefore, changes in the innervating neurons and the physiology of urothelium are associated with diabetic bladder dysfunction.

The pathophysiology of DUA in our diabetic DUA rat model is presumed to be myogenic. DUA pathology developed 21 days after induction of DM, with increased smooth muscle atrophy and apoptosis leading to decreased detrusor contractility, while little differences were observed in the neurofilaments and urothelium. The significant decrease in detrusor volume in our diabetic model might result in over-expression of receptors associated with detrusor contraction to maintain contraction (Figure 8b–d); however, alteration in the function of these receptors should be evaluated further. In addition, the *Ngf* transcript level was downregulated in our diabetic model but was not restored to a significant level in one week after M-MSC transplantation (Figure 8a). Reduced NGF production in the bladder or a deficiency in NGF transport to the bladder afferent pathway are observed in patients and animal models with diabetic cystopathy. In their study on rats with STZ-induced DM, Katsumi Sasaki et al. found a significant time dependent decrease in NGF levels in the bladder and in L6–S1 dorsal root ganglia (DRG) that was associated with voiding dysfunction, attributable to defects in δ and C-fiber bladder afferents [56]. Therefore, reduced NGF production in the bladder and/or impaired transport of NGF to L6 to S1 DRG, which contains bladder afferent neurons, could be an important mechanism underlying the induction of diabetic bladder dysfunction [54,55,56]. Therefore, limited recovery of nerves or *Ngf* expression in present study might be related to the limited long-term efficacy of M-MSC therapy.

According to the histological results, our diabetic DUA rat model was characterized by increased infiltration of mast cells in the bladder, which was significantly prevented by M-MSC transplantation (Figure 4a,c). A recent study employing a mouse ex vivo bladder afferent nerve recording, retrograde tracing of bladder-innervating DRG neurons, and in vivo spinal cord signaling assays revealed that histamine, an inflammatory mast cell mediator, enhances the sensitivity of bladder afferents to distension via interaction with histamine H1 receptor and transient receptor potential vanilloid-1 [53]. Sustained hyperglycemia resulted in oxidative stress and inflammation, which increased the mast cells and histamine production. However, as DM leads to damage in the peripheral neurons of the bladder, the effect of increased mechanosensitivity by the inflammatory mast cell mediator might have been neutralized. Taken together, further studies such as investigating the changes in peripheral nerves including DRG and urothelial physiology or inflammation are required to elucidate whether the mode of action for M-MSC therapy in diabetic cystopathy could include myogenic regeneration as well as denervation secondary to autonomic neuropathy or urothelium restoration.

Acontractile bladder is defined as occurring when “the detrusor cannot contract during urodynamic studies resulting in prolonged bladder emptying within a normal time span,” and is generally grouped with DUA under the clinical diagnosis of UAB [57]. Awake cystometry on long-term follow-up of DM animals (2 and 4 weeks following transplantation) revealed severely decreased contractility of the detrusor, which resembled detrusor acontractility or detrusor areflexia. A longer duration of STZ-induced DM could have caused more severe bladder damage that was not responsive to transplantation of M-MSCs. Implementation of stem cell therapy should be considered prior to the development of advanced disease, which is less likely to be responsive to any therapeutic modality.

Previously, we demonstrated that transplanted M-MSCs replenish denuded urothelium in the early stages of disease and progressively contribute to perivascular cells in IC/BPS rat models [24,26]. The precise mechanism for restoration of bladder function in the present diabetic DUA model is unclear, but histological changes suggested that M-MSCs could have ameliorated smooth muscle apoptosis and differentiated into pericytes in vessel-like structures proximal to the muscular layer. The paracrine effects of M-MSCs integrated into the vasculature could promote regeneration of damaged myocytes, restoring detrusor contractility. However, the in-depth mechanisms of action for M-MSC therapy should be further investigated in crucial future studies.

The primary limitation of the present study is that only one diabetic animal model was used. Diabetes can be generally classified as type 1 diabetes (the inability to produce enough insulin due to loss of insulin-producing β-cells) and type 2 diabetes (non-response of peripheral tissues to insulin leading to impaired uptake of glucose from the bloodstream). STZ is toxic to pancreatic β-cells and induces type 1 diabetes. However, most DM patients have type 2 diabetes, which has multifactorial etiologies, including genetics, environment, diet, and autoimmune pathologies [58]. However, because hyperglycemia is the primary causative factor of DM complications, animal models of both type 1 and type 2 diabetes are extensively used for investigation of DM complications.

## 5. Conclusions

Effective therapeutic strategies for DUA are limited. Ttransplantation of M-MSCs into the bladders of DM animals alleviated DUA by integrating into pericytes, providing a favorable microenvironment for myogenic restoration in the DM rat model with detrusor muscle atrophy. In addition, M-MSCs more effectively restored bladder function than currently available MSC therapy. However, duration of the therapeutic effect of M-MSC transplantation was short-term, limited to under 2 weeks. Therefore, further investigations are required to further improve the functionality and therapeutic potential of MSC therapy and to successfully translate this preclinical study into clinical applications.

## Supplementary Materials

The following are available online at https://www.mdpi.com/2077-0383/9/9/2853/s1, Figure S1: Surface phenotype of M-MSCs and UC-MSCs. Figure S2: Immunostaining of caspase 7 protein in DM bladders. Figure S3: Long-term effect of M-MSC therapy on the expression of genes related to diabetic DUA.

## References

[1] Abrams P., Cardozo L., Fall M., Griffiths D., Rosier P., Ulmsten U., Van Kerrebroeck P., Victor A., Wein A. (2003). The standardisation of terminology in lower urinary tract function: Report from the standardisation sub-committee of the International Continence Society. Urology.

[2] Aldamanhori R., Chapple C.R. (2017). Underactive bladder, detrusor underactivity, definition, symptoms, epidemiology, etiopathogenesis, and risk factors. Curr. Opin. Urol..

[3] Gammie A., Kaper M., Dorrepaal C., Kos T., Abrams P. (2016). Signs and Symptoms of Detrusor Underactivity: An Analysis of Clinical Presentation and Urodynamic Tests from a Large Group of Patients Undergoing Pressure Flow Studies. Eur. Urol..

[4] Chapple C.R., Osman N.I., Birder L., van Koeveringe G.A., Oelke M., Nitti V.W., Drake M.J., Yamaguchi O., Abrams P., Smith P.P. (2015). The underactive bladder: A new clinical concept?. Eur. Urol..

[5] Osman N.I., Chapple C.R., Abrams P., Dmochowski R., Haab F., Nitti V., Koelbl H., van Kerrebroeck P., Wein A.J. (2014). Detrusor underactivity and the underactive bladder: A new clinical entity? A review of current terminology, definitions, epidemiology, aetiology, and diagnosis. Eur. Urol..

[6] Zimmet P., Alberti K.G., Magliano D.J., Bennett P.H. (2016). Diabetes mellitus statistics on prevalence and mortality: Facts and fallacies. Nat. Rev. Endocrinol..

[7] Yuan Z., Tang Z., He C., Tang W. (2015). Diabetic cystopathy: A review. J. Diabetes.

[8] Arrellano-Valdez F., Urrutia-Osorio M., Arroyo C., Elena S.-V. (2014). A comprehensive review of urologic complications in patients with diabetes. Springerplus.

[9] Liu G., Li M., Vasanji A., Daneshgari F. (2011). Temporal diabetes and diuresis-induced alteration of nerves and vasculature of the urinary bladder in the rat. BJU Int..

[10] Deli G., Bosnyak E., Pusch G., Komoly S., Feher G. (2013). Diabetic neuropathies: Diagnosis and management. Neuroendocrinology.

[11] Tarcan T., Rademakers K., Arlandis S., von Gontard A., van Koeveringe G.A., Abrams P. (2018). Do the definitions of the underactive bladder and detrusor underactivity help in managing patients: International Consultation on Incontinence Research Society (ICI-RS) Think Tank 2017?. Neurourol. Urodyn..

[12] Osman N.I., Esperto F., Chapple C.R. (2018). Detrusor Underactivity and the Underactive Bladder: A Systematic Review of Preclinical and Clinical Studies. Eur. Urol..

[13] Thomas A.W., Cannon A., Bartlett E., Ellis-Jones J., Abrams P. (2005). The natural history of lower urinary tract dysfunction in men: Minimum 10-year urodynamic follow-up of untreated bladder outlet obstruction. BJU Int..

[14] Kim A., Shin D.M., Choo M.S. (2016). Stem Cell Therapy for Interstitial Cystitis/Bladder Pain Syndrome. Curr. Urol. Rep..

[15] Shin J.H., Ryu C.M., Yu H.Y., Shin D.M., Choo M.S. (2020). Current and Future Directions of Stem Cell Therapy for Bladder Dysfunction. Stem Cell Rev. Rep..

[16] Kim J.H., Lee S.R., Song Y.S., Lee H.J. (2013). Stem cell therapy in bladder dysfunction: Where are we? And where do we have to go?. Biomed. Res. Int..

[17] Lee S., Lim J., Lee J.H., Ju H., Heo J., Kim Y.H., Kim S., Yu H.Y., Ryu C.M., Lee S.Y. (2020). Ascorbic Acid 2-Glucoside Stably Promotes the Primitiveness of Embryonic and Mesenchymal Stem Cells Through Ten-Eleven Translocation- and cAMP-Responsive Element-Binding Protein-1-Dependent Mechanisms. Antioxid. Redox Signal..

[18] Lim J., Heo J., Ju H., Shin J.W., Kim Y.H., Lee S., Yu H.Y., Ryu C.M., Yun H.D., Song S. (2020). Glutathione dynamics determine the therapeutic efficacy of mesenchymal stem cells for graft-versus-host disease via CREB1-NRF2 pathway. Sci. Adv..

[19] Kim Y., Jin H.J., Heo J., Ju H., Lee H.Y., Kim S., Lee S., Lim J., Jeong S.Y., Kwon J. (2018). Small hypoxia-primed mesenchymal stem cells attenuate graft-versus-host disease. Leukemia.

[20] Knaän-Shanzer S. (2014). Concise Review: The Immune Status of Mesenchymal Stem Cells and Its Relevance for Therapeutic Application. Stem Cells.

[21] Hong K.S., Bae D., Choi Y., Kang S.W., Moon S.H., Lee H.T., Chung H.M. (2015). A porous membrane-mediated isolation of mesenchymal stem cells from human embryonic stem cells. Tissue Eng. Part C Methods.

[22] Kate E.H., Michelangelo C., Kate D., Anna M.R., Filipa V., Kwan L.H., Avina H., Donald P., Pierre G., Henrik H. (2018). Embryonic Stem Cell-Derived Mesenchymal Stem Cells (MSCs) Have a Superior Neuroprotective Capacity over Fetal MSCs in the Hypoxic-Ischemic Mouse Brain. Stem Cells Transl. Med..

[23] Sheyn D., Ben-David S., Shapiro G., Mel S.D., Bez M., Ornelas L., Sahabian A., Sareen D., Da X., Pelled G. (2016). Human Induced Pluripotent Stem Cells Differentiate into Functional Mesenchymal Stem Cells and Repair Bone Defects. Stem Cells Transl. Med..

[24] Kim A., Yu H.Y., Lim J., Ryu C.M., Kim Y.H., Heo J., Han Y.H., Lee H., Bae Y.S., Kim J.Y. (2017). Improved efficacy and in vivo cellular properties of human embryonic stem cell derivative in a preclinical model of bladder pain syndrome. Sci. Rep..

[25] Lee S.W., Ryu C.M., Shin J.H., Choi D., Kim A., Yu H.Y., Han J.Y., Lee H.Y., Lim J., Kim Y.H. (2018). The Therapeutic Effect of Human Embryonic Stem Cell-Derived Multipotent Mesenchymal Stem Cells on Chemical-Induced Cystitis in Rats. Int. Neurourol. J..

[26] Ryu C.M.Y.H., Lee H.Y., Shin J.H., Lee S., Ju H., Paulson B., Lee S., Kim S., Lim J., Heo J. (2018). Longitudinal intravital imaging of transplanted mesenchymal stem cells elucidates their functional integration and therapeutic potency in an animal model of interstitial cystitis/bladder pain syndrome. Theranostics.

[27] Lim J., Lee S., Ju H., Kim Y.H., Heo J., Lee H.Y., Choi K.C., Son J., Oh Y.M., Kim I.G. (2017). Valproic acid enforces the priming effect of sphingosine-1 phosphate on human mesenchymal stem cells. Int. J. Mol. Med..

[28] Heo J., Lim J., Lee S., Jeong J., Kang H., Kim Y.H., Kang J.K., Yu H.Y., Jeong E.M., Kim K. (2017). Sirt1 Regulates DNA Methylation and Differentiation Potential of Embryonic Stem Cells by Antagonizing Dnmt3l. Cell Rep..

[29] Heo J., Noh B.J., Lee S., Lee H.Y., Kim Y.H., Lim J., Ju H., Yu H.Y., Ryu C.-M., Lee P.C.W. (2020). Phosphorylation of TFCP2L1 by CDK1 is required for stem cell pluripotency and bladder carcinogenesis. EMBO Mol. Med..

[30] Jeong E.M., Yoon J.H., Lim J., Shin J.W., Cho A.Y., Heo J., Lee K.B., Lee J.H., Kim H.J., Son Y.H. (2018). Real-Time Monitoring of Glutathione in Living Cells Reveals that High Glutathione Levels Are Required to Maintain Stem Cell Function. Stem Cell Rep..

[31] Jeong E.M., Shin J.W., Lim J., Kim J.H., Kang H., Yin Y., Kim H.M., Kim Y.H., Kim S.G., Kang H.S. (2019). Monitoring Glutathione Dynamics and Heterogeneity in Living Stem Cells. Int. J. Stem Cells.

[32] Wu W.R., Lin J.T., Pan C.T., Chan T.C., Liu C.L., Wu W.J., Sheu J.J.-C., Yeh B.W., Huang S.K., Jhung J.Y. (2020). Amplification-driven BCL6-suppressed cytostasis is mediated by transrepression of FOXO3 and post-translational modifications of FOXO3 in urinary bladder urothelial carcinoma. Theranostics.

[33] Duan L., Ying G., Danzer B., Perez R.E., Madar Z.S., Levenson V.V., Maki C.G. (2014). The prolyl peptidases PRCP/PREP regulate IRS-1 stability critical for rapamycin-induced feedback activation of PI3K and AKT. J. Biol. Chem..

[34] Sadakata T., Kakegawa W., Mizoguchi A., Washida N., Katoh-Semba R., Shutoh F., Okamoto T., Nakashima H., Kimura K., Tanaka M. (2007). Impaired cerebellar development and function in mice lacking CAPS2, a protein involved in neurotrophin release. J. Neurosci..

[35] Varland S., Myklebust L.M., Goksoyr S.O., Glomnes N., Torsvik J., Varhaug J.E., Arnesen T. (2018). Identification of an alternatively spliced nuclear isoform of human N-terminal acetyltransferase Naa30. Gene.

[36] Zhang H., Qiu X., Shindel A.W., Ning H., Ferretti L., Jin X., Lin G., Lin C.-S., Lue T.F. (2012). Adipose tissue-derived stem cells ameliorate diabetic bladder dysfunction in a type II diabetic rat model. Stem Cells Dev..

[37] Jin Y., Xu L., Zhao Y., Wang M., Jin X., Zhang H. (2017). Endogenous Stem Cells Were Recruited by Defocused Low-Energy Shock Wave in Treating Diabetic Bladder Dysfunction. Stem Cell Rev..

[38] Thomson J.A., Itskovitz-Eldor J., Shapiro S.S., Waknitz M.A., Swiergiel J.J., Marshall V.S., Jones J.M. (1998). Embryonic stem cell lines derived from human blastocysts. Science.

[39] Itskovitz-Eldor J., Schuldiner M., Karsenti D., Eden A., Yanuka O., Amit M., Soreq H., Benvenisty N. (2000). Differentiation of human embryonic stem cells into embryoid bodies compromising the three embryonic germ layers. Mol. Med..

[40] Cho S.J., Kim S.Y., Jeong H.C., Cheong H., Kim D., Park S.J., Choi J.J., Kim H., Chung H.M., Moon S.H. (2015). Repair of Ischemic Injury by Pluripotent Stem Cell Based Cell Therapy without Teratoma through Selective Photosensitivity. Stem Cell Rep..

[41] Schwartz S.D., Regillo C.D., Lam B.L., Eliott D., Rosenfeld P.J., Gregori N.Z., Hubschman J.P., Davis J.L., Heilwell G., Spirn M. (2015). Human embryonic stem cell-derived retinal pigment epithelium in patients with age-related macular degeneration and Stargardt’s macular dystrophy: Follow-up of two open-label phase 1/2 studies. Lancet.

[42] Schwartz S.D., Hubschman J.P., Heilwell G., Franco-Cardenas V., Pan C.K., Ostrick R.M., Mickunas E., Gay R., Klimanskaya I., Lanza R. (2012). Embryonic stem cell trials for macular degeneration: A preliminary report. Lancet.

[43] Jurisicova A., Casper R.F., MacLusky N.J., Mills G.B., Librach C.L. (1996). HLA-G expression during preimplantation human embryo development. Proc. Natl. Acad. Sci. USA.

[44] de Almeida P.E., Ransohoff J.D., Nahid A., Wu J.C. (2013). Immunogenicity of pluripotent stem cells and their derivatives. Circ. Res..

[45] Zhang Y., Liang X., Liao S., Wang W., Wang J., Li X., Ding Y., Liang Y., Gao F., Yang M. (2015). Potent Paracrine Effects of human induced Pluripotent Stem Cell-derived Mesenchymal Stem Cells Attenuate Doxorubicin-induced Cardiomyopathy. Sci. Rep..

[46] Chin C.J., Li S., Corselli M., Casero D., Zhu Y., He C.B., Hardy R., Péault B., Crooks G.M. (2018). Transcriptionally and Functionally Distinct Mesenchymal Subpopulations Are Generated from Human Pluripotent Stem Cells. Stem Cell Rep..

[47] Liu G., Daneshgari F. (2014). Diabetic bladder dysfunction. Chin. Med. J. (Engl.).

[48] Wittig L., Carlson K.V., Andrews J.M., Crump R.T., Baverstock R.J. (2018). Diabetic bladder dysfunction: A review. Urology.

[49] Van Koeveringe G.A., Vahabi B., Andersson K.E., Herrmans R.K., Oelke M. (2011). Detrusor underactivity: A plea for new approaches to a common bladder dysfunction. Neurourol. Urodyn..

[50] Cucchi A., Quaglini S., Rovereto B. (2008). Development of idiopathic detrusor underactivity in women: From isolated decrease in contraction velocity to obvious impairment of voiding function. Urology.

[51] Osman N.I., Chapple C.R. (2014). Contemporary concepts in the aetiopathogenesis of detrusor underactivity. Nat. Rev. Urol..

[52] Osman N., Mangera A., Hillary C., Inman R., Chapple C. (2016). The underactive bladder: Detection and diagnosis. F1000Research.

[53] Grundy L., Caldwell A., Garcia Caraballo S.G., Erickson A., Schober G., Castro J., Harrington A.M., Brierley S.M. (2020). Histamine induces peripheral and central hypersensitivity to bladder distension via the histamine H1 receptor and TRPV1. Am. J. Physiol. Renal. Physiol..

[54] Tyagi P., Smith P.P., Kuchel G.A., de Groat W.C., Birder L.A., Chermansky C.J., Adam R.A., Tse V., Chancellor M.B., Yoshimura N. (2014). Pathophysiology and animal modeling of underactive bladder. Int. Urol. Nephrol..

[55] Yoshimura N., Chancellor M.B., Andersson K.E., Christ G.J. (2005). Recent advances in understanding the biology of diabetes-associated bladder complications and novel therapy. BJU Int..

[56] Sasaki K., Chancellor M.B., Phelan M.W., Yokoyama T., Fraser M.O., Seki S., Kubo K., Kumon H., de Groat W.C., Yoshimura N. (2002). Diabetic cystopathy correlates with a long-term decrease in nerve growth factor levels in the bladder and lumbosacral dorsal root Ganglia. J. Urol..

[57] Colaco M., Osman N.I., Karakeci A., Artibani W., Andersson K.-E., Badlani G.H. (2018). Current concepts of the acontractile bladder. BJU Int..

[58] Furman B.L. (2015). Streptozotocin-Induced Diabetic Models in Mice and Rats. Curr. Protoc. Pharmacol..

