# Supplementary figures and images for: Therapeutic Efficacy of Human Embryonic Stem Cell-Derived Multipotent Stem/Stromal Cells in Diabetic Detrusor Underactivity: A Preclinical Study
Source: J Clin Med. 2020 Sep 3;9(9):2853. doi: 10.3390/jcm9092853 (PMC7563486; doi:10.3390/jcm9092853)

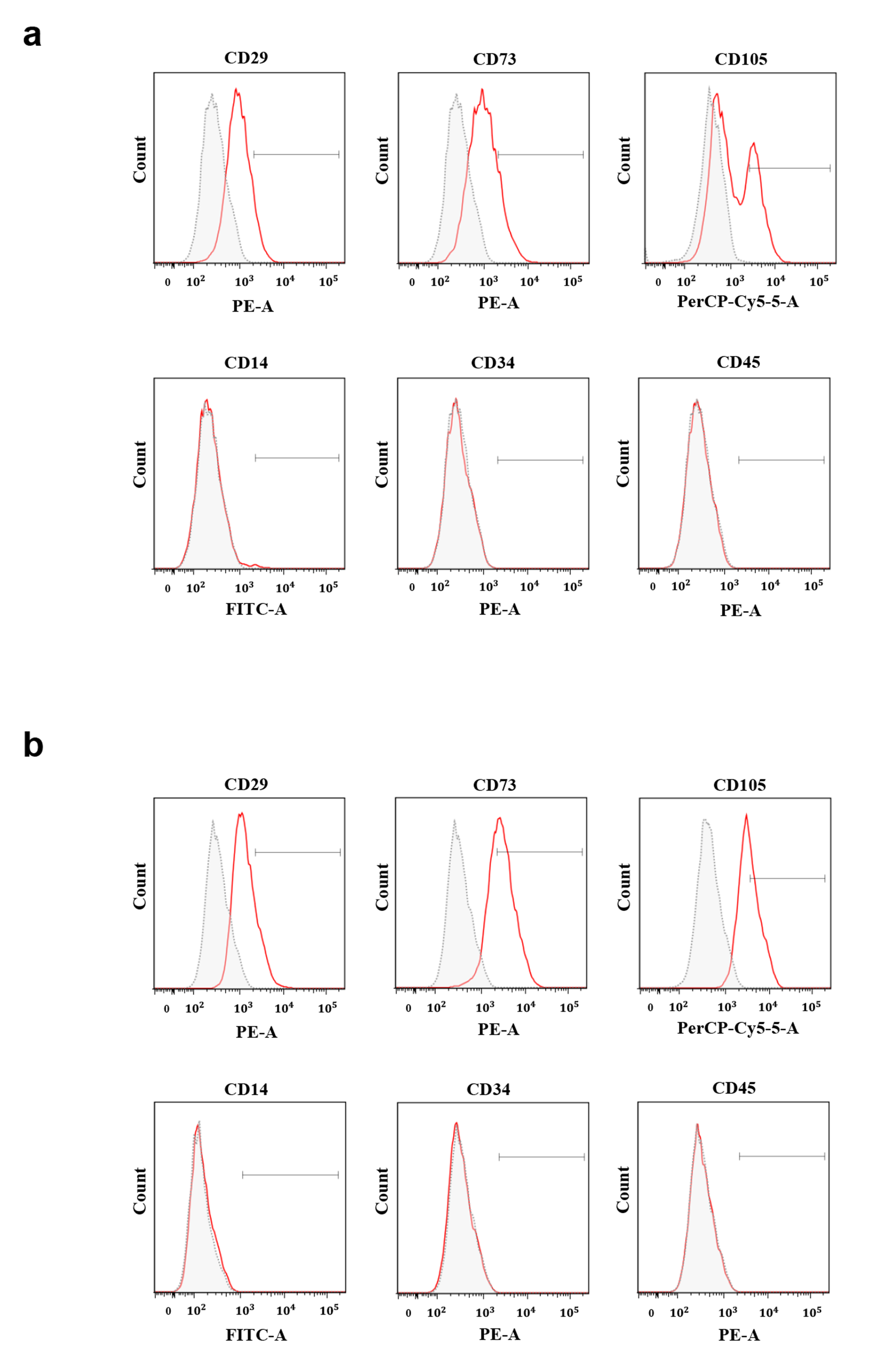

Supplement: Supplementary file 1 [file jcm-09-02853-s001.zip › JCM_Figure_SI_01_600dpi.tif]

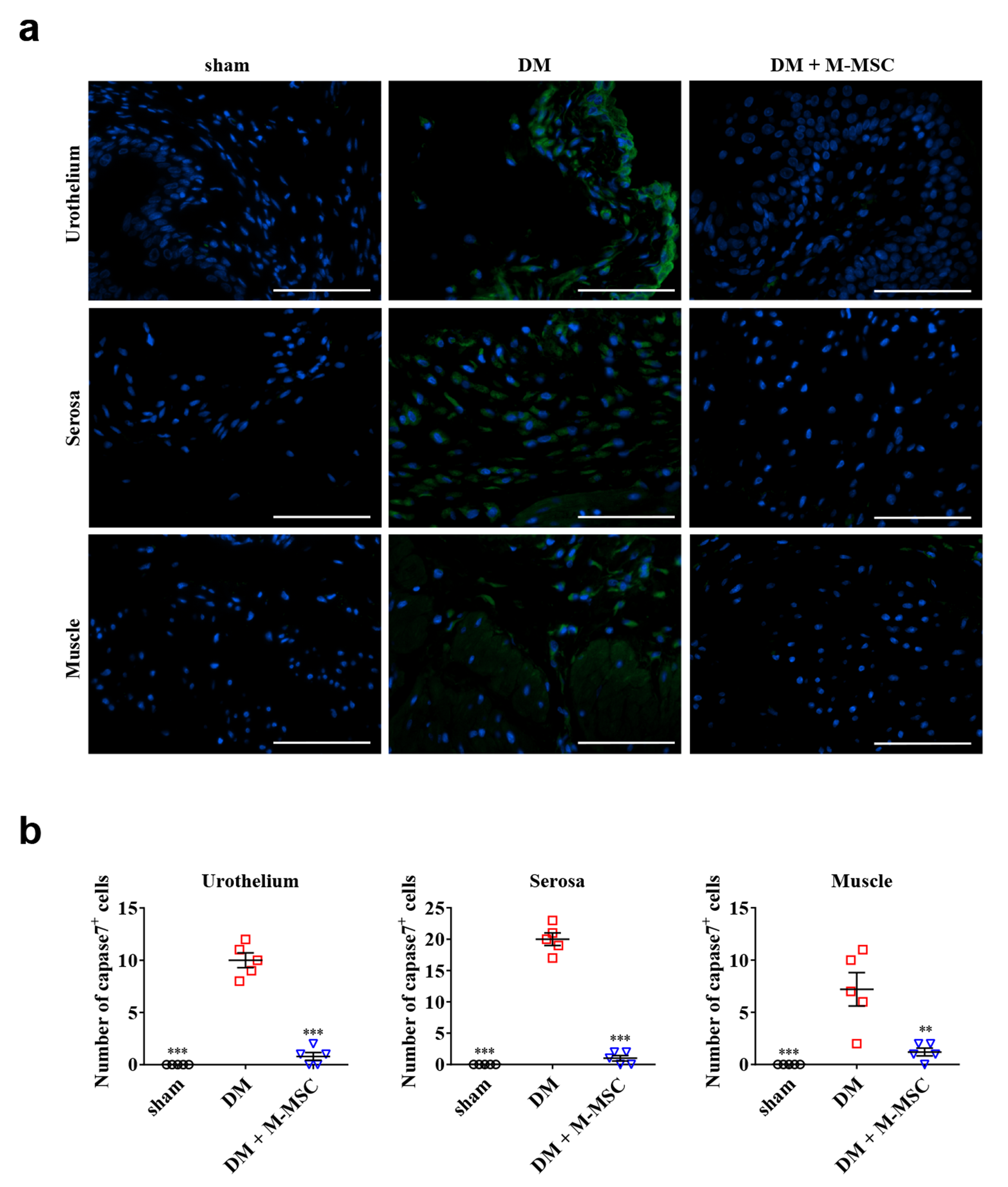

Supplement: Supplementary file 1 [file jcm-09-02853-s001.zip › JCM_Figure_SI_02_600dpi.tif]

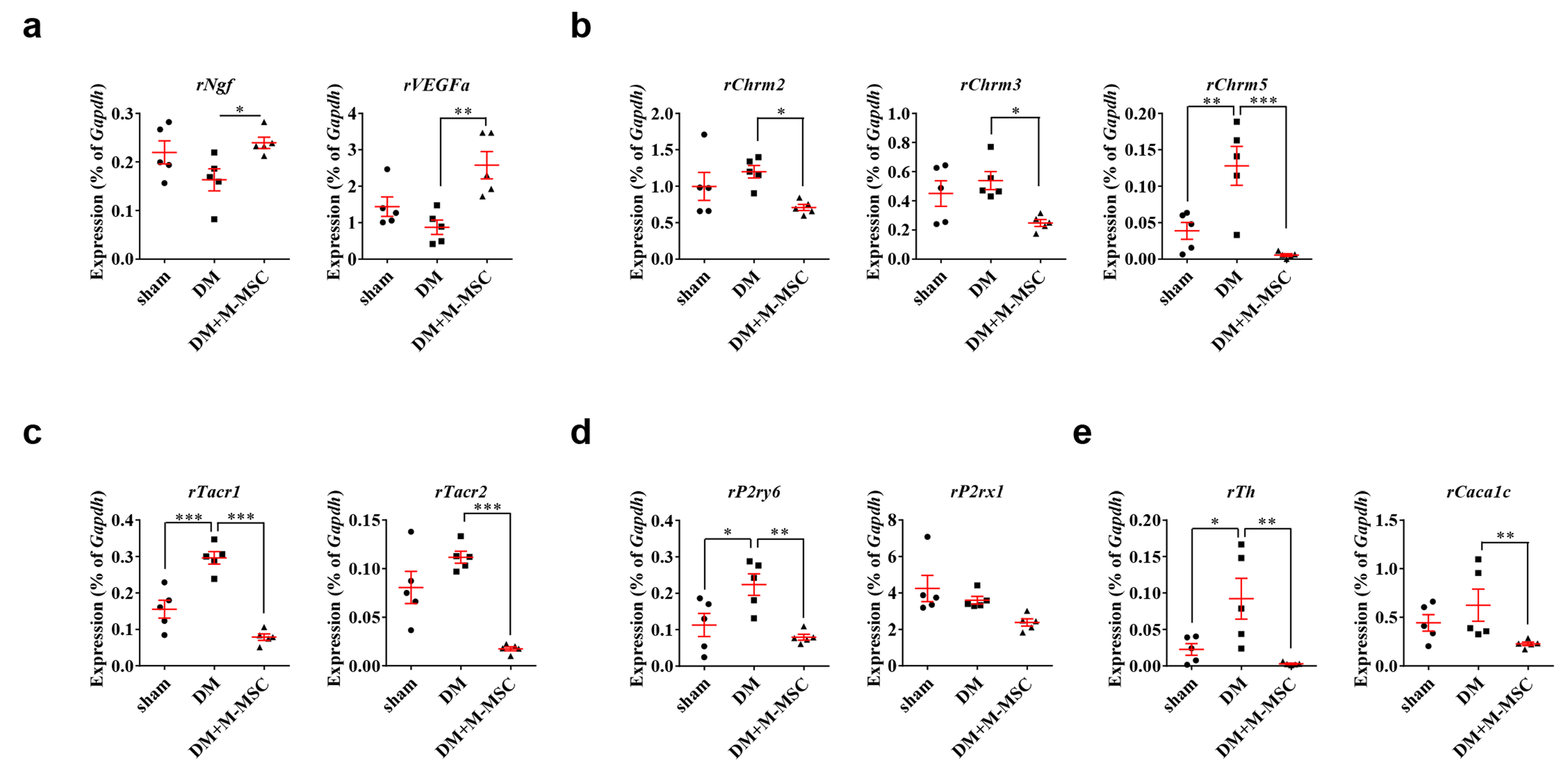

Supplement: Supplementary file 1 [file jcm-09-02853-s001.zip › JCM_Figure_SI_03_600dpi.tif]
