# Supplementary material for: Therapeutic Efficacy of Human Embryonic Stem Cell-Derived Multipotent Stem/Stromal Cells in Diabetic Detrusor Underactivity: A Preclinical Study
Source: J Clin Med. 2020 Sep 3;9(9):2853. doi: 10.3390/jcm9092853 (PMC7563486; doi:10.3390/jcm9092853)
Supplement: Supplementary file 1 [file jcm-09-02853-s001.zip › Shinetal_R1_Response.docx]

**Response to Reviewer #1. We thank the reviewer for carefully evaluating our paper. The reviewer raised some concerns, which we have addressed below in a point-by-point fashion.**

***Reviewer 1***

*The study by Jung Hyun Shin et al entitled ‘Therapeutic efficacy of human embryonic stem-cell derived multipotent stem cells in diabetic detrusor underactivity’ was designed to test the hypothesis that mesenchymal stem cells can be employed as a novel treatment for detrusor underactivity.*

*Over all the authors have designed a study of high quality that is of interest both scientifically and clinically. The authors provide a complex set of experiments to answer their research question, however there are a number of issues that significantly reduce the quality of the manuscript and the ability to correctly interpret the data presented.*

*Major comments*

*Q1. The focus of this study is on the detrusor smooth muscle as the cause of detrusor underactivity and employed a previously employed STZ induced model of DM. In corroboration with previous reports, the authors provide sufficient evidence to suggest that there are structural changes in the bladder following STZ induced DM. However, the authors should also introduce and discuss more thoroughly the known effect of DM on promoting neuropathy of bladder innervating neurons https://www.ncbi.nlm.nih.gov/pmc/articles/PMC4469340/, as well as the potential integrative role of the urothelium as has been outlined and thoroughly reviewed previously https://doi.org/10.1111/j.1464-410X.2005.05392.x. Many of the effects observed both in the model of DM and the effects of M-MSC have the potential to be derived from alterations to both the autonomic and sensory innervation of the bladder and should be comprehensively discussed.*

**Response: Thank you for this important comment. We agree with your point that changes in innervating neurons and loss of the integration role of urothelium might be associated with diabetic bladder dysfunction. In the present study, nerve growth factor (NGF) expression was downregulated in the diabetic model, but was not restored to a significant level after injecting multipotent mesenchymal stem/stromal stem cells (M-MSCs). This limited recovery of nerves might be related to the limited long-term efficacy of M-MSC therapy. Following your valuable comment, we are planning to focus on peripheral nerves including the dorsal root ganglion (DRG) and functional urothelium in further studies.**

**As per your suggestion, we have mentioned these points in the Discussion section of the revised manuscript (page 18, lines 10 – page 20, line 2) and have cited the attached references.**

*Q2. For all cystometry data, individual values should be presented to increase data transparency. For figures 1, 2, 3, and 5 it is currently unclear if there are 5 data points per group representing an average of each parameter for an individual mouse, or* *if multiple data points representing each micturition cycle are presented. The poor resolution of raw data images for the cytometry is a major limitation and needs to be resolved prior to publication.*

**Response: This is an important comment. In the original manuscript, multiple data points representing each micturition cycle were presented in the cystometry data. In the revised paper, to ensure data transparency, we used mean values from three reproducible voiding cycles for each individual animal when analyzing the cystometry data. This is clearly described in the Methods section of the revised manuscript (page 8, lines 13 – 14). Furthermore, we presented the Source data with the exact experimental values, number of replicates, and statistical test results as the Supplementary Table. Please see the revised manuscript and Supplementary Table**

*Q3. The representative traces in Figures 1, 2, 3, and 5 do not accurately reflect the group data. For example, Figure 1: The cystometric trace shows 4 micturition cycles over 8 minutes giving a micturition interval of approximately 120 seconds. The data in Figure 1b provides micturition intervals of approximately 50 seconds, with 0 seconds SEM. This does not fit with the data presented. Similarly for the DM group, the cystometric trace shows 2 micturition cycles over 8 minutes giving a micturition interval of approximately 240 seconds but really there is around 6 minutes (300s) between the two micturitions. The data in Figure 1b provides micturition intervals of approximately 100 seconds, with close to 0 seconds SEM. This does not fit with the data presented.*

**Response: For the quantification of cystometry data, the micturition interval (MI) was defined as the interval between micturition contraction cycles. As shown in the diagram below, this indicates the interval from the time resting to basal pressure in the preceding micturition cycle to the time rising from basal pressure in the following cycle.**

**We have modified the Methods section to describe this point better in the revised manuscript (page 8, lines 21 – 22):**

***“****Consistent with prior studies, the micturition interval (MI) was defined as the interval between micturition contraction cycles, representing the times between resting to basal pressure in the preceding micturition cycle and rising from basal pressure in the following cycle.”*

*Q4. The data presented in Figure 1C is presented as g/g which I assume is g of force contraction divided by g of tissue. Please clarify why raw data is not plotted.*

**Response: In accordance with the reviewer’s comment, we have modified the Methods section to describe this point better in the revised manuscript (page 11, line 1):**

***“****All tissue responses (g) were normalized to tissue weight (g tissue) for the analysis (g/g tissue).”*

*Q5. Please include the basal pressure data from Figure 2?*

**Response: As per the reviewer’s comment, we have included the basal pressure data in the revised Figure 2.**

*Q6. It is not clear what comparisons are being made for statistical significance in all figures, all columns should be compared to all other columns and statistical significance should be presented.*

**Response: As described in the Methods section, statistical significance was assessed using one-way or two-way ANOVA followed by Bonferroni’s post-hoc tests. Details of comparison groups were clearly described in each Figure legend. To increase transparency, we have presented the Source data with the exact experimental values, number of replicates, and statistical test results in the Supplementary Table. Please see the revised Supplementary Table.**

*Q7. Poor resolution of images for figure 4 makes it impossible to see toluidine staining.*

**Response: Thank you for this comment. We have changed the representative pictures for toluidine blue staining to pictures with a higher resolution in the revised Figure 4A as per your suggestion.**

*Q8. There is an increase in mast cells in the bladders of DM rats, yet there is a decrease in bladder voiding. Recent studies have shown that histamine, a major product of mast cells, is able to enhance bladder mechanosensitivity which in healthy states would lead to increased sensation an voiding https://doi.org/10.1152/ajprenal.00435.2019. The authors should discuss this with reference to peripheral neuropathy and autonomic bladder function.*

**Response: We thank the reviewer for this insightful comment. In the attached reference article, the authors evaluated the role of histamine in interstitial cystitis and bladder pain syndrome. Increased mast cells and their main product might have resulted in mechanosensitivity to bladder distension. This is a very interesting point in combination with your fist major comment. Sustained hyperglycemia resulted in oxidative stress and inflammation which increased mast the cells and histamine production. However, as diabetes also damaged peripheral neurons of the bladder, the effect of increased mechnosensitivity by histamine might have been neutralized. In future studies, we will investigate the role of histamine production and its associated receptors on the patho-physiology of diabetic cystopathy.**

**To clarify this point, we have described this point in the Discussion section of the revised manuscript (page 19, lines 13 – page 20, line 2) and have cited the attached reference.**

*Q9. The standard practice for analysis of qPCR data is via the double data Ct method. It is not clear what %Gapdh represents in figures 7 and 8 and the method of statistical analysis is not explained in the materials and methods section. The double data Ct method of analysis should be employed and data re-plotted.*

**Response: To better present the actual expression levels of the indicated genes, we used the percentage of *Gapdh* instead of relative values by the 2^-ΔΔCt^ method. We have modified the Methods section to better describe the procedures of our quantitative gene expression analysis in the revised manuscript (page 10, lines 12 – 16):**

***“****The threshold cycle (Ct) of the indicated genes was subtracted from that of Gapdh, an endogenous control gene, and the expression level of the target genes was quantified as 100 % × 2^−ΔCt(target-Gapdh)^, representing the percentage of Gapdh. Duplicate RQ-PCR assays were performed using five separate animals per group and the mean values from each individual animal were used for the analysis.”*

**In addition, we presented the Source data with the exact experimental values, number of replicates, and statistical test results in the Supplementary Table to ensure data transparency. Please see the revised Supplementary Table.**

*Q10. Based on the expression analysis of muscarinic receptors, there is significant upregulation of cholinergic receptors. As these receptors are known to be responsible for contraction of the detrusor, promoting efficient micturition, could the authors discuss how this fits with their cystometry data showing bladder underactivity and reduced EFS and carbachol concentration response curves.*

**Response: We thank the reviewer for this important comment. We assume that cholinergic receptors show compensatory upregulation due to their decreased function. As detrusor volume is significantly decreased in the diabetic model, the receptors associated with detrusor contraction might be overexpressed to maintain contraction. It might thus be interesting to evaluate receptor function in future studies.**

**We have clarified this point in the Discussion section of the revised manuscript (page 18, lines 22 – page 19, line 1):**

***“****The significant decrease in detrusor volume in our diabetic model might result in over-expression of receptors associated with detrusor contraction to maintain contraction (****Figure 8b−d****); however, alteration in the function of these receptors should be evaluated further.”*

*Minor comments*

*Q1. What was the volume of stem cells injected into the bladder?*

**Response: We apologize for this error. The injection volume of M-MSCs was 200 μL, as described in the revised manuscript (page 7, line 17):**

*“diabetic rats that received a single administration of the indicated dosage of human ESC-derived M-MSCs (0.25, 0.5, and 1×10^6^ cells; DM groups + 250 K, 500 K, 1000 K) resuspended in 200 μL phosphate buffered saline (PBS).”*

--------------------------------------------------------------------------

**Response to Reviewer #2. We greatly appreciate the reviewer’s general positivity and careful evaluation of our paper.**

***Reviewer 2***

*This is a very extensive study on the effect of multipotent MSCs (M-MSCs) on detrusor underactivity. Several points desserve, however, additional comments from the authors.*

*Q1. Authors should present the changes in Bladder Voiding Efficiency as this parameter may be used as a non-invasive parameter to detect detrusor underactivity.*

**Response: In accordance with the reviewer’s comment, the bladder voiding efficiency (BVE) has been quantified as 100 × ratio of the voided volume to the total bladder volume (micturition volume/bladder capacity; MV/BC). We have included the BVE results in the revised Figure 1b (for short-term effects) and Figure 2b (for long-term follow-up). To clarify this point, we have included the quantification method and results in the revised manuscript (page 8, lines 23 – 24; page 12, lines 1 – 2; page 12, lines 19 – 20).**

*Q2. Did all the animals have a similar response? Please show the results in dot plots.*

**Response: We thank the reviewer for this important suggestion. The other reviewer was also concerned about individual variation within the experimental group. To address this issue, we have updated the quantification data using the mean values from each animal (five individual animals per group) in the cystometry assay as well as the histological and gene expression examinations. As discussed in our response to comment 2 and 9 by Reviewer #1, we have modified the Methods section to describe our quantitative methods better in the revised manuscript (page 8, lines 13 – 14; page 10, line 2; page 10, lines 12 – 16).**

**As suggested, we have presented the all the quantitative data as dot plots in the revised Figures. Furthermore, we have presented the Source data with the exact experimental values, number of replicates, and statistical test results in the Supplementary Table to ensure data transparency. Please see the revised manuscript, Figures, and Supplementary Table.**

*Q3. Could the effect seen in this study be induced by growth factors or other proteins present in the culture medium for the M-MSCs? Which volume of culture medium was injected?*

**Response: As described in the Methods and Discussion, the trophic factors or growth factors secreted from M-MSCs integrated into the vasculature could induce beneficial paracrine effects by stimulating the regeneration of damaged myocytes, thus restoring detrusor contractility. However, the present study has limitations regarding the lack of in-depth mechanisms of action underlying the effects of M-MSC therapy. Therefore, this point should be investigated further in future studies. We have mentioned this in the revised manuscript (page 20, lines 17 – 20).**

**As discussed in our response to minor comment 1 by Reviewer #1, we injected the M-MSCs resuspended in 200 μL phosphate buffered saline (PBS).**

*Q4. The disappearance of collagen and reappearance of smooth cells is possible until certain stages of changes associated with underactivity. By using the same time of diabetes mellitus in the experiments, authors cannot say when the effect of treatment is no longer possible. Please comment.*

**Response: As shown in Figure 2, awake cystometry on long-term follow-up of DM animals (2 and 4 weeks following transplantation) revealed severely decreased contractility of the detrusor resembling detrusor acontractility or detrusor areflexia. A longer duration of STZ-induced DM could cause more severe bladder damage that might not be responsive to M-MSC transplantation. Unfortunately, we have not examined whether M-MSC therapy could be feasible after the development of advanced disease. It is thus crucial to consider the responsiveness of the MSC-based therapeutic modality in clinical situations. Therefore, further longitudinal studies are required to investigate the therapeutic efficacy of M-MSCs in diabetic DUA with several experimental animals in which the extent of DM is well characterized. We thank the reviewer for this instructive comment.**

*Q5. One aspect that is not discussed is the change in NGF. However, it may have a very important effect in nerve survival, in particular sensory fibers.*

**Response: We thank the reviewer for this important comment. The other reviewer expressed a similar concern about the lack of discussion for the neurogenic aspects in the present study. In their study on rats with streptozotocin induced diabetes mellitus (DM) Katsumi Sasaki et al. found a significant time dependent decrease in NGF levels in the bladder and in L6–S1 DRG that was associated with voiding dysfunction, attributable to defects in δ and C-fiber bladder afferents (Sasaki et al, J Urol, 2002, 168(3), p1259-64). Therefore, the reduced NGF production in the bladder and/or impaired transport of NGF to L6 to S1 DRG, which contains bladder afferent neurons, could be an important mechanism underlying the induction of diabetic bladder dysfunction. Unfortunately, NGF was not significantly restored after M-MSC therapy in the present study. Therefore, further investigation on neurons including the dorsal root ganglion in diabetes model seems necessary.**

**As discussed in our response to major comment 1 by Reviewer #1, we have mentioned these points in the Discussion section of the revised manuscript , to clarify this issue (page 18, lines 10 – page 19, line 12). We thank the reviewer for this important comment.**

*Q6. Can authors comment about changes in the urothelium?*

**Response: This is an important point. The biology of DM-associated bladder complications is multifactorial and is characterized by the combined results of alteration in the physiology of the detrusor smooth muscle cell, innervation or function of the neuronal component, or the urothelial dysfunction. The urothelium may have receptors and ion channels similar to those in bladder nerves and the release of many neurotransmitter mediators such as adenosine triphosphate, nitric oxide, and prostanoids from urothelial cells can be aggravated by urothelial injury or inflammation. In our diabetic DUA rat model, minimal differences were observed in the urothelium; thus, we have not investigated the integration function of the urothelium. However, the exact role of the urothelium in M-MSC therapy for diabetic DUA cannot be excluded and should be investigated in a further study.**

**To clarify this point, we have described this issue in the Discussion section of the revised manuscript (page 18, lines 10 – 22). We thank the reviewer for this insightful comment.**

-----------------------------------------------------------------------------------------

**Response to Reviewer #3. We thank the reviewer for praising the significance of our study.**

*Reviewer 3*

*Authors examined the therapeutic efficacy of cells derived from human ESCs in DM-associated detrusor underactivity. All results are clear and well described. They showed that single local transplantation of cells ameliorated DUA bladder pathology, including functional changes and histological evaluation, and caused few adverse outcomes. Immunostaining and gene expression analysis revealed that the transplanted cells supported myogenic restoration primarily by engrafting into bladder tissue via pericytes and subsequently exerting paracrine effects to prevent apoptotic cell death in bladder tissue. The therapeutic efficacy of multipotent mesenchymal stromal cells was superior to that of human umbilical cord-derived stromal cells at the early time point.*

*Comments*

*Q1. I would recommend changing title and abbreviation (M-MSCs) from multipotent mesenchymal stem cells to multipotent mesenchymal stromal cells according to the International Society for Cellular Therapy (ISCT) guidelines. The same for UC-MSCs... More here: Nature 561, 455-457 (2018), DOI: 10.1038/d41586-018-06756-9*

**Response: In accordance with the reviewer’s suggestion, we have changed the title and abbreviation of “mesenchymal stem cells” into “mesenchymal stem/stromal cells” throughout the revised manuscript.**

*Q2. How was M-MSCs/UM-MSCs phenotype confirmed? Sacchetti et al. (2016) reported that cells from different tissues, all classed as MSCs, differ in their gene expression and differentiation potential.*

**Response: This is an important point. To address this, we examined the surface phenotypes of the M-MSCs and UC-MSCs used in this study by flow cytometry analysis. As shown in the revised Supplementary Figure 1, both MSCs expressed CD29, CD73, and CD105 surface molecules, but lack CD14, CD34, and CD45 hematopoietic lineage markers, thus confirming the surface phenotypes characteristic to MSCs. We have presented these results as the revised Supplementary Figure 1 and have described them in the Methods section of the revised manuscript (page 6, lines 18 – 21). We thank the reviewer for this important comment.**

---------------------------------------------------------------------------------

**Response to Reviewer #4. We greatly appreciate the reviewer’s generally positive review and careful evaluation of our paper.**

***Reviewer 4***

*Detrusor underactivity is a common clinical problem and lacks effective therapeutic strategies. Therefore, new approaches are desired and required. In this manuscript, the authors tackled the problem using mesenchymal stem cell therapy. The topic here is interesting in the research field of stem cell biology and therapy for detrusor underactivity.*

*Comments*

*Q1. The authors used different types of stem cells in this study. To characterize the stem cells used in this study, the authors should perform western blot or immunofluorescence analysis using proper stem cell markers.*

**Response: We thank the reviewer for this important comment. Other reviewers have expressed a similar concern. As discussed in our responses to comment 2 by Reviewer #3, we showed that the M-MSCs and UC-MSCs used in this study showed the surface phenotypes characteristic to typical MSCs by flow cytometry analysis. We have presented these results as the revised Supplementary Figure 1. Please see the revised Supplementary Figure 1.**

*Q2. In Fig. 8, the authors examined the effects of M-MSC therapy on the expression of genes related to diabetic DUA using bladders of DM rats at 1 week after injection of M-MSCs or PBS. To evaluate the usefulness of the stem cell therapy in the long term, the author should show the same data using DM rats at 4 weeks after injection of M-MSCs or PBS.*

**Response: We thank the reviewer for this insightful comment. As shown in Figure 2, M-MSC transplantation did not substantially restore the DUA voiding phenotypes in either the 2- or 4-week long-term follow-ups. In this regard, we did not examine the effect of M-MSC therapy on the expression of genes related to diabetic DUA in the previous draft. In accordance with the reviewer’s suggestion, the expression level of the genes was examined in the bladders of the animals at 4 weeks after M-MSC transplantation. As shown in the revised Supplementary Figure 3, the alteration of gene expression was still observed in the majority of genes related to diabetic DUA and M-MSC transplantation significantly restored these changes induced by diabetic DUA, indicating the long-term effects of M-MSC therapy on gene expression. The discrepancy between cystometry and gene expression analysis for the long-term effect of M-MSC therapy should be further investigated to understand its precise mode of action.**

**To clarify this point, we have presented these results in the revised Supplementary Figure 3 and have described them in the Results section of the revised manuscript (page 16, lines 5 – 7). We thank the reviewer for this insightful comment.**

*Q3. Page 2, line 52, there is an extra space between myogenic and dysfunction.*

**Response: We apologize for this mistake and have corrected it.**

**--------------------------------------------------------------------------------------------------------**

**In addition to the responses to Reviewers’ comments, we have added the results for immunofluorescence staining of caspase 7 protein in Supplementary Figure 2.**

**We believe that this extensively revised version of our manuscript is clear and scientifically strengthened. We hope it now meets with the reviewers’ approval for publication in the *Journal of Clinical Medicine*.**
